# Paclitaxel Resistance and Multicellular Spheroid Formation Are Induced by Kallikrein-Related Peptidase 4 in Serous Ovarian Cancer Cells in an Ascites Mimicking Microenvironment

**DOI:** 10.1371/journal.pone.0057056

**Published:** 2013-02-25

**Authors:** Ying Dong, Carson Stephens, Carina Walpole, Joakim E. Swedberg, Glen M. Boyle, Peter G. Parsons, Michael A. McGuckin, Jonathan M. Harris, Judith A. Clements

**Affiliations:** 1 Cancer Program, Institute of Health and Biomedical Innovation and Faculty of Sciences and Technology, Queensland University of Technology, Kelvin Grove, Queensland, Australia; 2 Drug Discovery Group, Division of Cancer and Cell Biology, Queensland Institute of Medical Research, Herston, Queensland, Australia; 3 Immunity, Infection and Inflammation Program, Mater Medical Research Institute, South Brisbane, Queensland, Australia; University of Patras, Greece

## Abstract

High tumor kallikrein-related-peptidase 4 (KLK4) levels are associated with a poor outcome for women with serous epithelial ovarian cancer (EOC), for which peritoneal dissemination and chemoresistance are key events. To determine the role of KLK4 in these events, we examined KLK4-transfected SKOV-3 and endogenous KLK4 expressing OVCA432 cells in 3-dimensional (3D) suspension culture to mimic the ascites microenvironment. KLK4-SKOV-3 cells formed multicellular aggregates (MCAs) as seen in ascites, as did SKOV-3 cells treated with active KLK4. MCA formation was reduced by treatment with a KLK4 blocking antibody or the selective active site KLK4 sunflower trypsin inhibitor (SFTI-FCQR). KLK4-MCAs formed larger cancer cell foci in mesothelial cell monolayers than those formed by vector and native SKOV-3 cells, suggesting KLK4-MCAs are highly invasive in the peritoneal microenvironment. A high level of KLK4 is expressed by ascitic EOC cells compared to matched primary tumor cells, further supporting its role in the ascitic microenvironment. Interestingly, KLK4 transfected SKOV-3 cells expressed high levels of the KLK4 substrate, urokinase plasminogen activator (uPA), particularly in 3D-suspension, and high levels of both KLK4 and uPA were observed in patient cells taken from ascites. Importantly, the KLK4-MCAs were paclitaxel resistant which was reversed by SFTI-FCQR and to a lesser degree by the general serine protease inhibitor, Aprotinin, suggesting that in addition to uPA, other as yet unidentified substrates of KLK4 must be involved. Nonetheless, these data suggest that KLK4 inhibition, in conjunction with paclitaxel, may improve the outcome for women with serous epithelial ovarian cancer and high KLK4 levels in their tumors.

## Introduction

Serous epithelial ovarian carcinoma (EOC) accounts for >50% of ovarian cancer [1] which is the leading cause of death from gynecological malignancies [2]. Approximately 75% of women with EOC are diagnosed when tumors have spread into the peritoneal cavity [3] and ∼70% of these patients accumulate ascites [4]. Distinct from other solid tumors, EOC metastasis occurs as the tumor cells are shed from the primary site and form multicellular aggregates (MCAs) in the 3-dimensional (3D)-suspension ascites microenvironment, before adhering to the peritoneal surface and establishing secondary tumors in the underlying extracellular matrix (ECM) [5].

Survival in the ascites microenvironment is crucial for EOC cells to succeed in peritoneal dissemination. Although the underlying mechanism is not clear, it is known that the ascitic EOC cells are biologically different from their counterparts in the solid matrices of primary and metastatic sites [6]. For example, cell adhesion proteins E-cadherin [7] and α5/αv/β1 integrins [8] are highly expressed in ascites-derived ovarian cancer cells compared to those from primary or metastatic tumor sites. Of note, the serine protease, urokinase plasminogen activator (uPA) and its receptor uPAR in EOC cells are induced by ascites [9] and the expression of uPA is associated with chemoresistance, progression and poor prognosis in women with this cancer [10], [11]. These studies indicated that the tumor microenvironment clearly influences EOC progression [12], [13], but the effect of suspension per se, thus mimicking the ascites microenvironment, on survival of EOC cells and chemosensitivity is not clear. In particular, the involvement of other serine proteases remains largely unknown.

The kallikrein-related-peptidase (KLK) family comprises 15 serine peptidases that have shown their potential as biomarkers in human cancers [14], [15], [16]. These peptidases degrade ECM proteins and activate growth factors and other proteases, such as the uPA/uPAR axis [17], [18], that play a role in human cancer progression [14], [15], [16]. In ovarian cancer, KLK4-KLK8, KLK10 and KLK14 are upregulated [14], [15], [19], [20] and we previously reported that KLK4 and KLK7 were highly expressed in the most lethal histotype, serous EOCs [21], [22]. Recently, we showed that high *KLK7* levels are associated with poor prognosis and chemoresistance in women with serous EOC and that KLK7 induces MCA of SKOV-3 cells, most likely through an integrin related mechanism [23]. Given that high KLK4 levels are also reported to be associated with poor prognosis [24] and chemoresistance [25], in this study, we aimed to determine whether a similar, perhaps KLK-specific, functional mechanism was occurring. We show here that, like for KLK7, KLK4-over-expression in SKOV-3 cells promotes MCA formation and paclitaxel resistance in 3D-suspension cultures that mimic the ascites microenvironment, but unlike that seen in KLK7-SKOV-3 cells we found no association with integrin expression. However, KLK4 overexpressing SKOV-3 cells displayed upregulated levels of uPA, particularly in 3D-suspension MCAs. Importantly, KLK4 inhibition reduced MCA compaction and increased paclitaxel sensitivity in KLK4-MCAs. This data suggests that although several KLKs are over-expressed in EOC and may be similarly associated with EOC progression, the underlying mechanism of action will be related to the specific selective enzyme specificity of each KLK peptidase.

## Materials and Methods

### Materials

Antibodies used include those against V5 epitope tagged at the C-terminal of KLK4 (Invitrogen, Mount Waverley, VIC, Australia); a KLK4 catalytic-domain antibody, KLK4 functional blocking antibody (R&D Systems, Bio-Scientific Pty. Ltd, Gymea, NSW, Australia); monoclonal anti-uPA B-chain (American Diagnostica, Stamford, CT, USA); GAPDH and an anti-mouse IgG (Sigma Aldrich Pty Ltd, Castle Hill, NSW, Australia). Mouse and rabbit Alexa Fluor 488 secondary antibodies, Alexa Fluor 568 phalloidin and CellTracker^492^ were from Invitrogen. The generation of active recombinant KLK4 [26] and the selective active site KLK4 sunflower trypsin inhibitor (SFTI-FCQR) [27] are as published. Site-directed mutagenesis was used to generate the catalytic triad serine to alanine mutant-KLK4S207A (KLK4S/A) plasmid. All other chemicals were from Sigma except where noted.

### Human Cell Lines, and Patient Serous EOC Biopsies and Ovarian Tissue RNA

The SKOV-3 serous EOC and LP9 peritoneal mesothelial cell lines were from American Type Culture Collection and Coriell Cell Repositories respectively. The OVCA432 cell line was established from ascites obtained from an EOC patient [28] and is a generous gift from Dr. Samuel Mok (MD Anderson Cancer Center, Houston, TX, USA). The origin of patient EOC cells is described previously [21], [22]. The serous EOC tissue RNA samples were described previously [23]. Patient clinical information was obtained from Royal Brisbane and Women’s Hospital (Supplementary Table S1). Ethical approval was obtained from institutional ethics committees (Human Research Ethic Committee of Queensland University of Technology (#0800000213) and The Clinical and Statewide Services Research Committee (#229)); written consent was obtained from all patients.

### RNA Extraction, Reverse Transcription-PCR (RT-PCR)

Total RNA extraction and synthesis of cDNA are described previously [23]. Quantitative-RT-PCR was performed for 40 cycles on an ABI7300 thermal cycler (Applied Biosystems, Mulgrave, VIC, Australia) using *KLK4* specific primers (K4Ex2qS, 5′-GGCACTGGTCATGGAAAACGA-3′ and K4Ex3qAS, 5′-TCAAGACTGTGCAGGCCCAGCC-3′) and SYBR green as per manufacturer’s instructions. *KLK4* expression was normalized to *18S* (18SFor, 5′- GATCCATTGGAGGGCAAGTCT-3′ and 18SRev, 5′-CCAAGATCCAACTACGAGCTTTTT-3′) using the standard curve method and RT-PCR was performed as previously described [23].

### Generation of Stable Cell Lines

Generation and transfection of the plasmid (pcDNA3.1/V5-His, C-terminal V5 tag, Invitrogen) expressing the wild-type pre-pro-KLK4 was described previously [29]. The catalytic triad serine to alanine mutant-KLK4S207A (KLK4S/A) plasmid was generated by site-directed mutagenesis. Stable monoclonal KLK4-expressing or vector control cells were selected using G418 (Invitrogen), and 3 over-expressing clones, KLK4-1, KLK4-2 and KLK4-3, were randomly selected for the following *in vitro* functional assays.

### In vitro Functional Assays

#### In vitro migration assays

2×10^5^ cells in RPMI-1640 containing 0.1% BSA were seeded in tissue culture inserts with 8 µm pores (BD Biosciences, Eight Mile Plains, QLD, Australia), and allowed to migrate towards 10% FCS as the chemoattractant in the lower chamber for 24 hours (h). The number of migrated cells was quantified using crystal violet staining read at 595 nm.

#### Multicellular aggregate (MCA)/spheroid formation and inhibition

The hanging-drop method [30] was used for MCA formation of all transfected and native cells with 5×10^3^ cells/well in the presence of 10% FCS RPMI-1640 (100 µl) on top of agarose-coated plates (60 µl of 0.5% agarose/serum-free media, w/v) and incubated at 37°C. When recombinant active KLK4 (rKLK4) enzyme and catalytic inactive mutant KLK4S/A (50 ng/ml) were used to induce MCA formation of SKOV-3 cells, this was performed under serum free conditions. Serum free RPMI-1640 was used for MCA inhibition with the KLK4 blocking antibody at a concentration (10 µg/ml) to capture all active enzyme with a mouse IgG (10 µg/ml) control. KLK4 active site sunflower trypsin inhibitor (SFTI-FCQR, 1 µM) [27] or PBS controls were added into 10% FCS RPMI-1640. Images were taken using a Nikon-Eclipse TE2000-U digital camera (4×objectives) and V++ software. Compact MCAs were defined as those with ≥30 µm diameter. To quantify the percentage of cells that formed compact MCAs (≥30 µm), all visible spheroids (<30 µm, ≥30 µm) were counted at all time points and were divided by the number at 4 h, the time point chosen to allow the cells to settle in the well. The difference of overall spheroid numbers and those with <30 µm diameters on day 1, 4 and 7 from 4 h was calculated and was considered the percentage of compact MCAs formed. This approach was based on a previous report by Iwanicki et al [31].

#### In vitro mesothelial clearance assay

LP9 mesothelial cells (5,000) were seeded in 96-well plates and grown to ∼80% confluence. MCAs were washed in PBS, incubated in CellTracker^492^ (4 µM), added on top of mesothelial monolayers (∼4–6 spheroids/well/200 µl) and cultured at 37°C. At 4 h, 1, 2, 3 and 7 days from the initial spheroid plating, images were taken with a 10×objective. To quantify MCA clearance, the diameter of the fluorescent areas of MCAs labeled with CellTracker^492^ was measured using InDesign software (Adobe, Adobe Systems Pty Ltd, Chatswood, NSW, Australia). Measurements were performed on 10 randomly selected MCAs from 3 separate experiments for the average diameter on day 3 in comparison to that of the initial area of the spheroid (4 h).

#### Cell survival post cisplatin/paclitaxel treatment

24 h after seeding in uncoated 96-well plates (Nunc) as 2D-monolayers, cells were treated with cisplatin (0, 1, 5, 10, 50 µM) or paclitaxel (0, 0.01, 0.1, 1, 10 nM). In 3D-suspension cultures, spheroids were formed as above for 4 days and then cisplatin or paclitaxel was added. For KLK4 inhibition in 3D-suspension, the KLK4-1 or OVCA432 cells were resuspended in SFTI-FCQR (1 µM) containing media and seeded, and on day 4 paclitaxel (0, 0.1, 1, 10, 50, 100 nM) was added. WST-1 assays were performed 96 h after treatment. Cell survival was calculated as the percentage of absorbance of non-treated cells.

### Knockdown of KLK4 Expression

Knockdown of KLK4 expression was performed as described previously [26]. Briefly, the mammalian siRNA expression vector pSilencer 3.1-H1 puro (Ambion, Austin, TX, USA) was used to reduce expression of KLK4. Candidate KLK4 siRNA target sequences were designed with the Ambion siRNA program and then aligned against the human genome data base using the BLAST algorithm to eliminate those with significant homology to other genes. Two sequences selected were 5′-GATCCATCCCTGGGGCTGGTTCCTTTCAAGAGAAGGAACCAGCCCCAGGGATTTTTTTGGAAA-3′ (psilK4Ex1) and 5′-GATCCAACGAATTGTTCTGCTCGGTTCAAGAGACCGAGC- AGAACAATTCGTTTTTTTTGGAAA-3′ (psilK4Ex2). The oligos were synthesized (Sigma) and inserted into the pSilencer 3.1-H1 puro vector (Ambion) according to the manufacturer’s instructions. SKOV-3 stably expressing KLK4 (KLK4-1 clone) cells were transfected with the KLK4 pSilencer 3.1-H1 constructs or the supplied pSilencer 3.1-H1 negative control using Lipofectamine (Invitrogen). After 48 h, whole cell lysate was collected from transfected cells and expression of KLK4 and uPA was examined by Western blot analysis.

### Western Blotting

Whole cell lysates from cells cultured as monolayer for 3 days were collected in a buffer containing Complete protease inhibitor cocktail (1×, Roche Applied Sciences, Castle Hill, NSW, Australia), 50 mM Tris-HCl (pH 7.4), NaCl (150 mM) and CHAPS (1%). Cells cultured in 3D-collagen I, 3D-Matrigel™ (BD Biosciences) and 3D-suspension for 7 days were collected into ice-cold PBS followed by the procedure above. Protein concentration was determined by the microbicinchoninic acid assay (Thermo Fisher Scientific, Scoresby, VIC, Australia). Cell lysates (20 µg) or conditioned media (CM, 4 µg protein) were separated by SDS-PAGE under reducing conditions, transferred to nitrocellulose membranes, and blocked in Odyssey blocking buffer (LI-COR® Biosciences, Lincoln, NE, USA). Membranes were incubated with primary antibodies diluted in blocking buffer overnight at 4°C, washed with tris-buffered saline containing 0.05% Tween-20, and then incubated with secondary IRDye 680 or 800 conjugated mouse or rabbit IgG (LI-COR® Biosciences) as appropriate. Images were generated and densitometry analysis was performed using the Odyssey system and software (LI-COR® Biosciences).

### Confocal Microscopy

Cells grown on sterile cover-slips until 80% confluent or MCAs collected in eppendorf tubes after 7 days culture were fixed (4% w/v paraformaldehyde/PFA in PBS), permeabilized (0.5% v/v triton X-100 in PBS) and blocked (5% w/v bovine serum albumin/BSA in PBS). Incubation with primary antibodies against KLK4 and E-Cadherin (1/200 v/v in 1% BSA in PBS) was at 4°C overnight. Alexa Fluor 568 phalloidin and 4′-6-diamidino-2-phenylindole (DAPI, Sigma) were applied with secondary Alexa Fluor 488 IgG as appropriate. To obtain the images of MCA clearance, monolayer mesothelial LP9 cells were seeded in an 8-chamber slide (In vitro Technologies, Noble Park North, VIC, Australia), CellTracker^492^ was applied for KLK4-1 spheroids prior to seeding and 24 h later, cells were fixed as above followed by staining of Alexa Fluor 568 phalloidin and DAPI; MCAs of Vector-1, SKOV-3 and OVCA432 were stained with E-cadherin as above. Images were taken using a Leica-TCS SP5 confocal microscope (63×and 20×oil immersion objective lens for monolayer cells and MCAs respectively) and associated software. Z section stacking images were generated using the Maximum Projection software with aspect ratio 2.

### Statistical Analysis

Student’s t-test was used for functional analyses with *P*≤0.05 considered to be significant. For survival analysis, patients were categorized into two groups with either low (n = 25) or high (n = 13) *KLK4* levels using a median cut-off of *KLK4* copy number, after normalization to *18S,* of 0.0007 (range 0.0000147 to 0.0258). Two different endpoints, cancer relapse and patient death were used to calculate post-surgery progression free survival (PFS) and overall survival time respectively. Kaplan-Meier analysis was used to determine the association of *KLK4* level with PFS and overall survival time by a log rank model. The analyses were performed using SPSS 18.0 for Windows (SPSS Inc, Chicago, IL, USA).

## Results

### KLK4-expressing SKOV-3 Cells are Less Migratory but are Chemoresistant as 2D Monolayers

**Figure 1:**
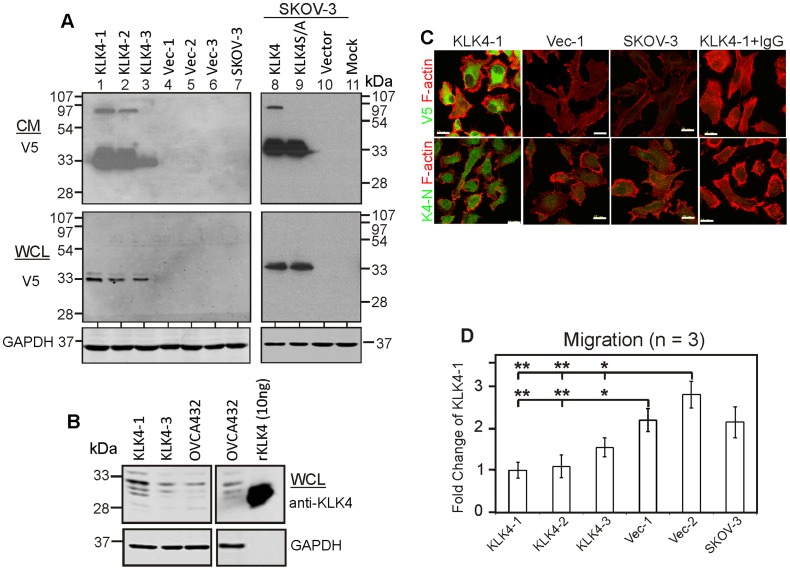
KLK4-expressing SKOV-3 cells are less migratory as 2D monolayers. **A.** Western blotting with anti-V5 antibody shows KLK4 expression in the conditioned media (CM) and whole cell lysate (WCL) from stable KLK4 tranfectants (KLK4-1, KLK4-2 and KLK-3; lanes 1–3), vector (Vec-1, Vec-2 and Vec-3; lanes 4–6) and native SKOV-3 (lane 7) cells, and transiently expressed wildtype KLK4, mutant-KLK4S207A (KLK4S/A), vector and mock transfectants (lanes 8–11); GAPDH was used as a loading control for WCL. **B.** Western blotting with anti-KLK4 antibody shows relative levels of KLK4 protein in WCL of KLK4-1, KLK4-3 clones and OVCA432, and 10 ng of recombinant (r)KLK4 protein. **C.** IF microscopy with anti-V5/KLK4-N terminal antibodies (green) and phalloidin (red) in KLK4-1, Vec-1 clones, native SKOV-3 or negative control (IgG). Scale bar, 20 µm. **D.** Transwell migration assays with KLK4-1, KLK4-2, KLK4-3, Vec-1, Vec-2 and native SKOV-3 cells; n = 3, mean ± SEM, *P<0.05.

We wished to determine the mechanism of action underlying the poor prognosis [24] and chemoresistance [25] previously reported to be associated with high *KLK4* levels in ovarian cancer generally. Thus, we generated both stable and transient transfectants in SKOV-3 cells that express little endogenous KLK4 for functional analysis (Supplementary Fig. S1A). Western blot analysis confirmed that the V5-tagged over-expressed KLK4 (33 kDa), an extracellular serine protease, is secreted in the conditioned media (CM) of both stable and transient KLK4 transfectants but not vector-only, mock-transfected or native SKOV-3 cells (Fig. 1A, top panel). Interestingly, an 88 kDa protein band was seen in the CM from two of the high KLK4-expressing clones (KLK4-1 and KLK4-2) and on transient transfection, but not the CM of a serine to alanine active site mutant-KLK4S207A (KLK4S/A) construct (Fig. 1A, top panel), or the whole cell lysate (WCL) from wild-type KLK4 or mutant KLK4S/A transfectants (Fig. 1A, bottom panel). These data indicate that both wild-type KLK4 and mutant KLK4S/A are secreted but only the wild-type KLK4 is present as an active enzyme as shown by the 88 kDa band which is likely KLK4 covalently bound with an unidentified serpin. To support this observation that KLK4 is activated and forms complexes with serum borne proteins or inhibitors, active KLK4 (100 or 500 nM) was incubated with culture medium (RPMI-1640), with or without 1% and 5% FCS for 2 and 18 h respectively. Western blot analysis shows that the 88 kDa species is present only when active KLK4 was incubated in RPMI-1640 media with FCS but not when FCS was absent (Supplementary Fig. S1B). The level of KLK4 expression in the KLK4-3 clone is comparable to that of endogenous KLK4 expressing OVCA432 cells while the KLK4-1 clone shows more intense KLK4 protein bands (Fig. 1B). Immunofluorescent (IF) microscopy using antibodies against the V5-tagged C-terminus and N-terminal peptide of KLK4 [29] further confirmed protein over-expression in each of the KLK4 clones, but negligible expression in vector control, native SKOV-3 cells or KLK4-1 IgG control (Fig. 1C, Supplementary Fig. S1C).

Previous *in vitro* studies have shown that KLK serine proteases cleave collagens I and IV, fibronectin, vitronectin and laminin [32], [33] that are components of the peritoneal membrane [34] and we reported that KLK7 over-expression increased adhesion to fibronectin and vitronectin [23]. In this study, however, we did not see any difference between the KLK4-transfected and control cells in adherence to these ECM components or proliferation (data not shown). On the other hand, KLK4 expressing clones showed less transwell migration compared to vector/native control cells (*P<0.05 or **P<0.01, Fig. 1D). In 2D-monolayer cultures, using concentrations (cisplatin 0–50 µM; paclitaxel 0–100 µM) within the range used for patient treatment (cisplatin 3–50 µM; paclitaxel 3–20 µM) [35], KLK4-transfected SKOV-3 cells were more resistant to cisplatin than vector/native control cells (*P<0.05, Supplementary Fig. S2, left panel), although only a trend towards paclitaxel resistance was seen for KLK4-transfected cells (Supplementary Fig. S2, right panel). These data suggest that any changes induced by KLK4 over-expression were relatively subtle and that cisplatin rather than paclitaxel resistance, as seen with KLK7 transfected SKOV-3 cells, would be the more clinically relevant phenotype.

### KLK4-expressing SKOV-3 Cells form Compact MCAs that Spread on Mesothelial Cell Monolayers

**Figure 2:**
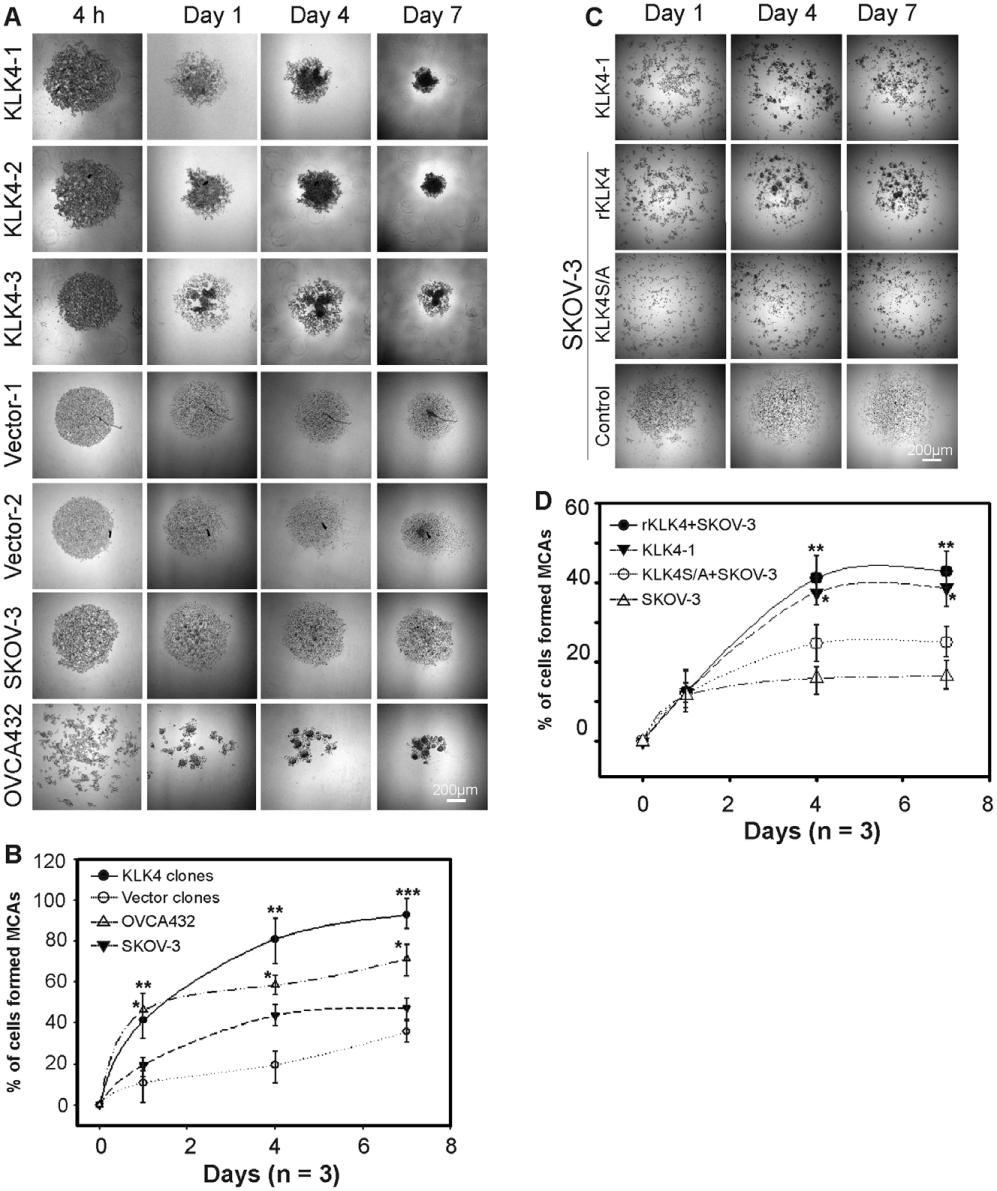
KLK4-expressing SKOV-3 cells form compact MCAs in a 3D-suspension microenvironment. **A.** MCA/spheroid formation conducted in 10% FCS containing media at 4 h, day 1, 4 and 7, with representative images of KLK4-1, KLK4-2, KLK4-3, Vec-1, Vec-2 clones, native SKOV-3 and endogenous KLK4 expressing OVCA432 cells. **B.** Quantitative analysis of percentage of 4 h (0 time point) that formed compact MCAs (≥30 µm) after 1, 4 and 7d from the 3 KLK4 clones combined, 2 vector clones combined, native SKOV-3 and OVCA432 cells. **C.** MCA formation under serum free conditions by KLK4-1 clone and native SKOV-3 cells treated with 50 ng/ml active recombinant (r)KLK4or mutant-KLK4S207A (KLK4S/A) and PBS control at day 1, 4 and 7. **D.** Quantitative analysis of compact MCA formation of KLK4-1, SKOV-3 treated with rKLK4, KLK4S/A and PBS as a control over 1, 4 and 7d. For panels **A** and **C**, scale bars, 200 µm; for **B** and **D**, the experiment was repeated 3 times with triplicates; mean ± SEM; *P<0.05; **P<0.01; and ***P<0.001.

Due to the role of homotypic cell adhesion for EOC cell survival in the 3D-suspension microenvironment of ascites [36] and our previous finding that KLK7 could induce spheroid formation [23], we compared the ability of KLK4 transfected and control cells to form MCAs/spheroids. By 1, 4 and 7 days post seeding, two of three KLK4 clones generated large compact MCAs with more cells while the lower KLK4-expressing KLK4-3 cells formed slightly less compact MCAs, with vector controls and native SKOV-3 cells forming small and scattered spheroids in 10% FCS containing media (Fig. 2A). In support of this finding, the endogenous KLK4 expressing OVCA432 cells formed more compact MCAs than SKOV-3 cells that had little KLK4. Quantitative analysis showed that KLK4-expressing cells formed more compact MCAs than vector control cells on day 1, 4 (**P<0.01) and 7 (***P<0.001, Fig. 2B), as did the endogenous KLK4 expressing OVCA432 cells compared to SKOV-3 cells (*P<0.05, Fig. 2B). As we have shown that some active KLK4 is bound to unknown binding proteins or serpins in serum containing media (Supplementary Fig. S1B) suggesting KLK4 enzymatic activity could be inhibited in the presence of serum, we used serum free conditions to test the addition of active KLK4. Under these conditions, the KLK4-1 cells formed less compact MCAs (Fig. 2C) than those seen in 10% FCS (Fig. 2A). Addition of recombinant active KLK4 (rKLK4, 50 ng/ml) induced native SKOV-3 cells to form MCAs more similar to that observed for the KLK4-1 clone whereas the mutant KLK4S/A (catalytically inactive, 50 ng/ml) treated SKOV-3 cells had a similar appearance to that of the SKOV-3 cells treated with PBS as a control (P<0.05, Fig. 2C), confirming the involvement of KLK4 activity in SKOV-3 cell aggregation. Quantitative analysis showed that active rKLK4 treated SKOV-3 cells formed compact spheroids at a similar rate to KLK4-1 cells, and both were greater than those treated with non-active mutant KLK4S/A (*P<0.01) which was slightly greater that that seen for the SKOV-3 control on day 4 and 7, (**P<0.01, Fig. 2D). These data confirmed the role of the KLK4 peptidase in MCA formation but also suggested a possible non-catalytic action of KLK4 in this process.

**Figure 3:**
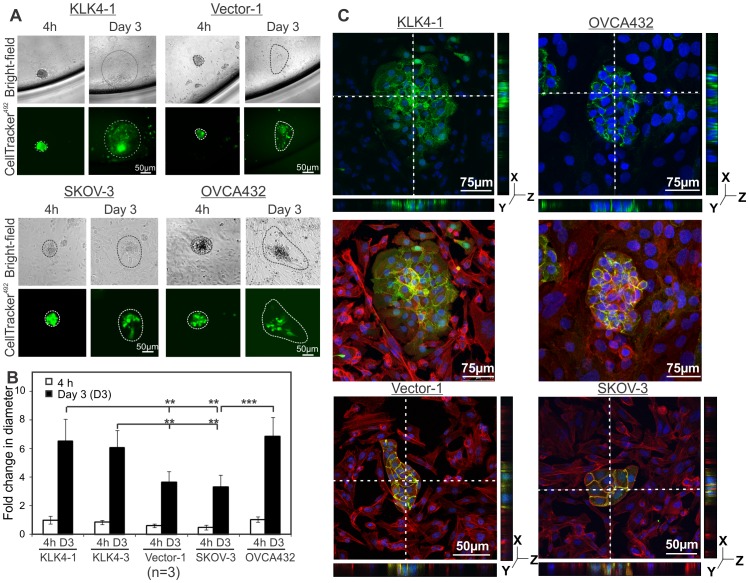
MCAs clear mesothelial monolayers mimicking invasion into the peritoneal membrane. **A.** Bright-field and fluorescence (CellTracker^492^) images show KLK4-1, Vector-1, SKOV-3 and OVCA432 MCA (4 h and day 3) clearance of mesothelial monolayers. Discontinuous lines indicate perimeters of the spreading MCAs. **B.** Quantitative analysis shows the average diameter of 10 MCAs from 3 separate experiments for above cell lines at 4 h and day 3 respectively; mean ± SE, n = 3, **P<0.01. **C.** IF microscopy images show mesothelial monolayer clearance of MCAs formed by KLK4-1 labeled with CellTracker^492^, Vector-1, OVCA432 and SKOV-3 cells stained with an E-cadherin antibody (green); both MCAs and mesothelial LP9 cells were stained with Phalloidin for F-actin (Alexa Flour 568, red) and DAPI for nuclei (blue) respectively; discontinuous lines indicate positions of multiple Z sections shown as right and bottom panels. For panels **A** and **C**, scale bars, 50 µm.

We then examined the invasiveness of KLK4-MCAs when added onto peritoneal mesothelial cell monolayers. Bright-field and fluorescent (CellTracker^492^) images show that the compact KLK4-1 MCA adhered to the live LP9 mesothelial monolayer and gradually formed an extensive front of spreading cancer cells over 3 days (Fig. 3A, top left panel) and up to 7 days (data not shown) confirming their viability and growth on the mesothelial monolayer. The MCAs formed by vector-1 control cells also spread across the mesothelial cell monolayer albeit to a less degree (Fig. 3A, top right panel). The size of the foci generated by the endogenously KLK4 expressing OVCA432 MCAs was similar to those of the transfected KLK4-1 MCAs (Fig. 3A, bottom panel). Quantitative analysis demonstrated that the diameter of the areas of fluorescent KLK4 MCA clearance (>6 fold) was larger than that of the Vector-1 clone (3.5 fold) on day 3 compared to their clonal controls at 4 h (**P<0.01, Fig. 3B). Similarly, the diameter of the KLK4 endogenously expressing OVCA432 MCA clearance on day 3 (7 fold compared to 4 h) was comparable to that of the KLK4-1 SKOV-3 clone while the native SKOV-3 MCA clearance (3.2 fold compared to 4 h) was comparable to the Vector transfected SKOV-3 cells (Fig. 3B). Confocal images show that CellTracker^492^ stained KLK4-1, and endogenously KLK4 expressing OVCA432 MCAs stained with E-cadherin grew into the mesothelial monolayers (Fig. 3C, top and middle panels) with multiple Z stacking images confirming the clearance of mesothelial monolayers by the MCAs to the bottom of the well (Fig. 3C, top panels). Vector-1 and SKOV-3 MCAs can also invade into the mesothelial monolayers but formed smaller invading foci due to fewer cell numbers respectively (Fig. 3C, bottom panels). These data suggest that the survival mechanism of cellular aggregation is induced by KLK4 in the 3D-suspension ascites mimicking microenvironment and that these KLK4 expressing spheroids will have an increased spreading capacity into the mesothelial layer of the peritoneum.

### KLK4 Inhibition Increased Paclitaxel Sensitivity

**Figure 4:**
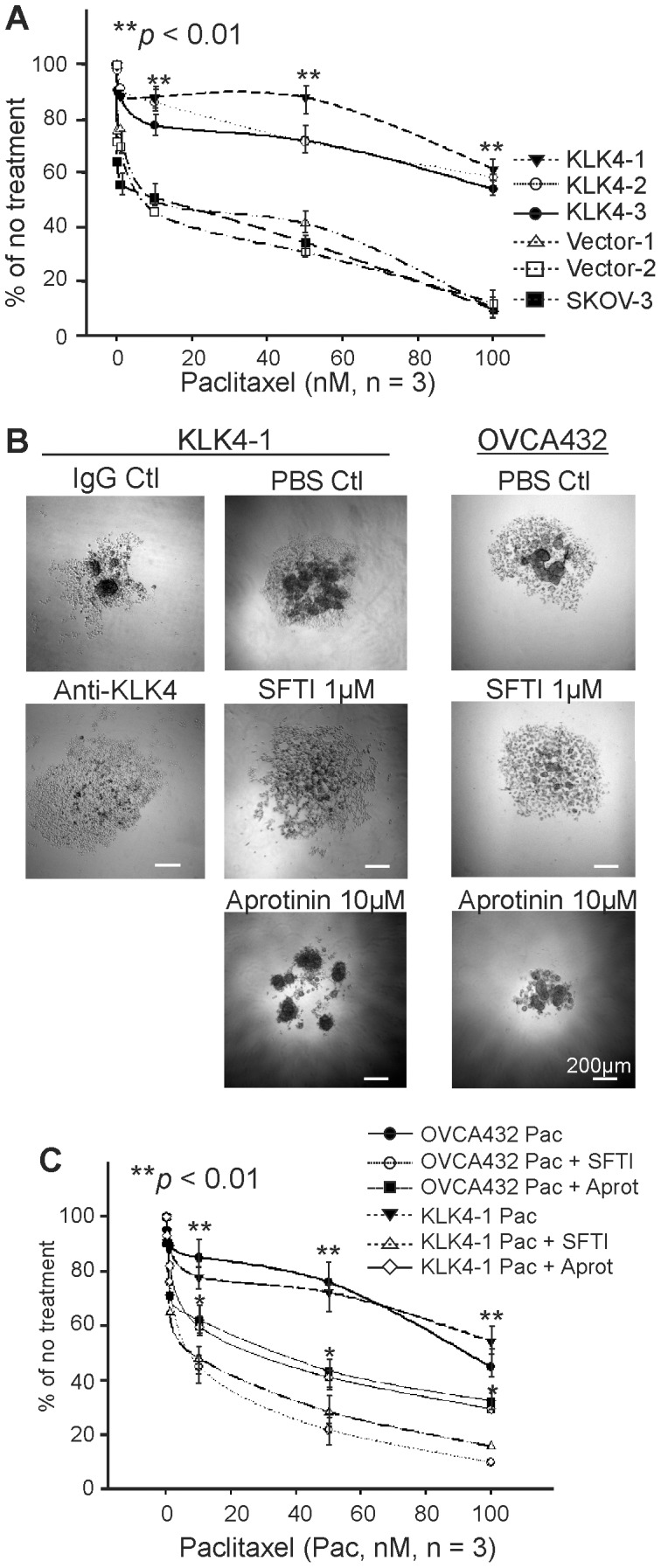
Inhibition of KLK4 increased paclitaxel sensitivity. WST-1 assay shows cell survival of KLK4-1, KLK4-2, KLK4-3, Vec-1, Vec-2 clones and native SKOV-3 MCAs after paclitaxel (**A**) treatment in 3D-suspension. **B.** Left panel, representative images of KLK4-1 MCAs on day 4 with mouse IgG and a functional KLK4 blocking antibody, PBS as a control and KLK4 selective inhibitor SFTI-FCQR (SFTI) as indicated. Right panel, representative images of OVCA432 MCAs on day 4 with PBS as a control and KLK4 selective inhibitor SFTI-FCQR (SFTI) or aprotinin as indicated. Scale bars, 200 µm. **C.** Cell survival determined by WST-1 assay after treatment with paclitaxel (Pac) on 3D-suspension cultured KLK4-1 clone and OVCA432 cells +/−1 µM SFTI or 5 µM aprotinin (Aprot). Experiments in panels **A** and **C** were repeated 3 times in triplicate, bars represent Mean ± SEM. Statistical significance indicated as *P<0.05, **P<0.01.

Although KLK4-transfected SKOV-3 cells were more resistant to cisplatin than vector/native control cells (Supplementary Fig. S2, left panel), no difference in cisplatin responsiveness was seen between KLK4 and vector/native control cells in 3D suspension (data not shown). In contrast, although only a trend towards paclitaxel resistance was seen for KLK4-transfected cells in 2D-monolayer (Supplementary Fig. S2, right panel), KLK4-MCAs in 3D-suspension were clearly more resistant to paclitaxel than vector/native cells (**P<0.01, Fig. 4A). Notably, compaction of KLK4-1 MCAs was reduced by adding a KLK4 blocking antibody at a concentration (10 µg/ml) to capture all active enzyme or the selective active site KLK4 sunflower trypsin inhibitor (SFTI-FCQR), compared to their appropriate controls (Fig. 4B, left and middle panels). Similarly, compaction of the endogenously expressing OVCA432 MCAs was reduced by adding SFTI-FCQR (Fig. 4B, right panel). In addition, although 1 µM SFTI-FCQR alone did not reduce proliferation (data not shown), it significantly reduced KLK4-1 and OVCA432 MCA survival on combined treatment with paclitaxel (**P<0.01, Fig. 4C). Interestingly, the general serine protease inhibitor, aprotinin, partially reduced the compaction of MCAs formed by KLK4-1 and OVCA432 cells (Fig. 4B, bottom panel) and increased their sensitivity to paclitaxel (*P<0.05, Fig. 4C), albeit to a lesser degree than SFTI-FCQR. These data emphasized that KLK4-MCAs are resistant to paclitaxel and reduced MCA compaction by KLK4 blockade increased sensitivity to this drug.

### KLK4 Induced uPA Expression in SKOV-3 Cells

**Figure 5:**
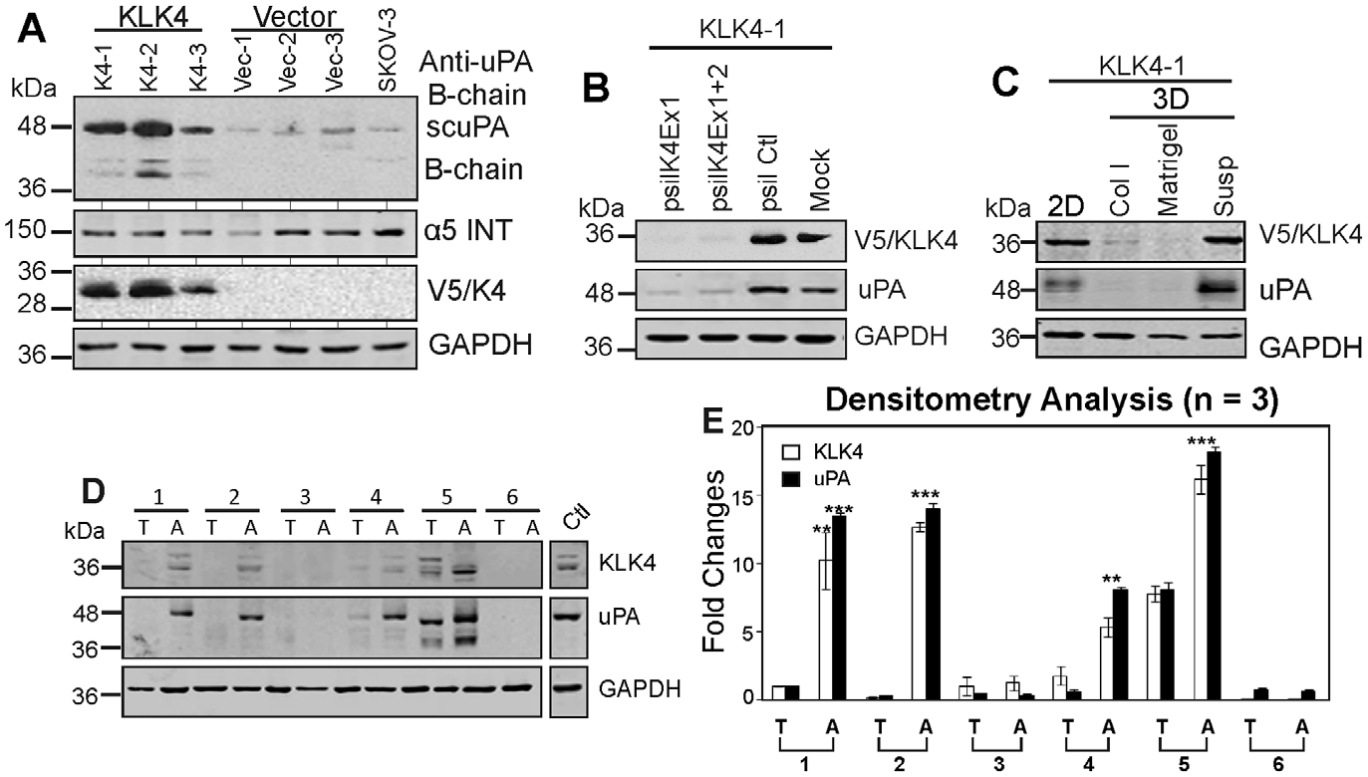
KLK4 induced uPA expression in SKOV-3 cells. **A.** Western blot analysis shows expression of uPA, α5 integrin (ITN) and KLK4 (V5) of 3 KLK4 and 3 vector control clones with native SKOV-3 cells as a control with GAPDH as a loading control. **B.** Western blot analysis shows expression of KLK4 and uPA in KLK4-1 clone transfected with siRNA KLK4 exon 1 (psilK4Ex1), and both KLK4 exon 1 and 2 knockdown constructs (psilK4Ex1+2), p-silencing scramble (psil Ctl) and mock controls. GAPDH was used as a loading control. **C.** Western blotting shows expression of KLK4 and uPA in KLK4-1 cells cultured as 2D-monolayers (2D), 3D-collagen I (Col I), 3D-Matrigel (Matrigel), and 3D-suspension (Susp), with GAPDH as a loading control. **D.** Western blot shows expression of KLK4 and uPA in serous EOC cells of primary tumors (T) and ascites (A) from 6 patients. WCL of OVCA432 MCAs serves as a positive control and GAPDH as a loading control. **E.** Densitometry analysis of 3 Western blots indicative of that shown in **D.** **P<0.05 and ***P<0.001 indicate the significantly different levels of KLK4 and uPA in ascitic (A) and primary tumor cells (T).

Given that aprotinin can also inhibit the action of other serine proteases, such as uPA that was activated by KLK4 in previous biochemical studies [17], [18], we examined the expression of uPA in KLK4 transfected SKOV-3 cells. We observed that whole cell lysates of KLK4 transfected SKOV-3 clones, KLK4-1, KLK4-2 and KLK4-3, displayed intense uPA bands at ∼48 and 38 kDa compared to vector and native SKOV-3 control cells on Western blot analysis (Fig. 5A). However, there is essentially no difference in protein band intensity of α5 (Fig. 5A) or β1 integrin (data not shown) observed in KLK4 clones compared to control cells. These data from KLK4 expressing cells are different from the upregulated expression of α5/β1 integrins observed in KLK7 transfected SKOV-3 cells [23], and underscore the different pathways modulated by each KLK family member. To examine if the increased uPA is induced by KLK4 over-expression, KLK4 knockdown was performed in the KLK4-1 transfected SKOV-3 cells. Western blotting on whole cell lysates from these cells showed that expression of uPA was also reduced (Fig. 5B), confirming that KLK4 over-expression induces uPA in SKOV-3 cells. Interestingly, expression of both KLK4 and uPA was only observed when KLK4-1 cells were cultured as a 2D-monolayer or in 3D-suspension, but not on 3D matrices of type I collagen or Matrigel (Fig. 5C), further suggesting a specific association of these two proteases with different 3D microenvironments. Importantly, Western blotting and densitometry analysis showed co-expression and significantly high protein levels of both KLK4 and uPA in ascitic EOC cells (4/6, Fig. 5D–E) compared to cells derived from the primary tumor of these patients. The slightly different molecular weight of these proteases in individual patient samples may reflect their post-translational modification, such as glycosylation of KLK4 as we and others have reported previously [29], [37]. These data suggest that the 3D-suspension microenvironment reprograms the expression of these proteases in EOC cells which is reflected in the differential expression of KLK4 and uPA as seen in ascitic compared to primary EOC cells.

### KLK4 is Associated with Poor Outcome of Patients with Serous EOCs

**Figure 6:**
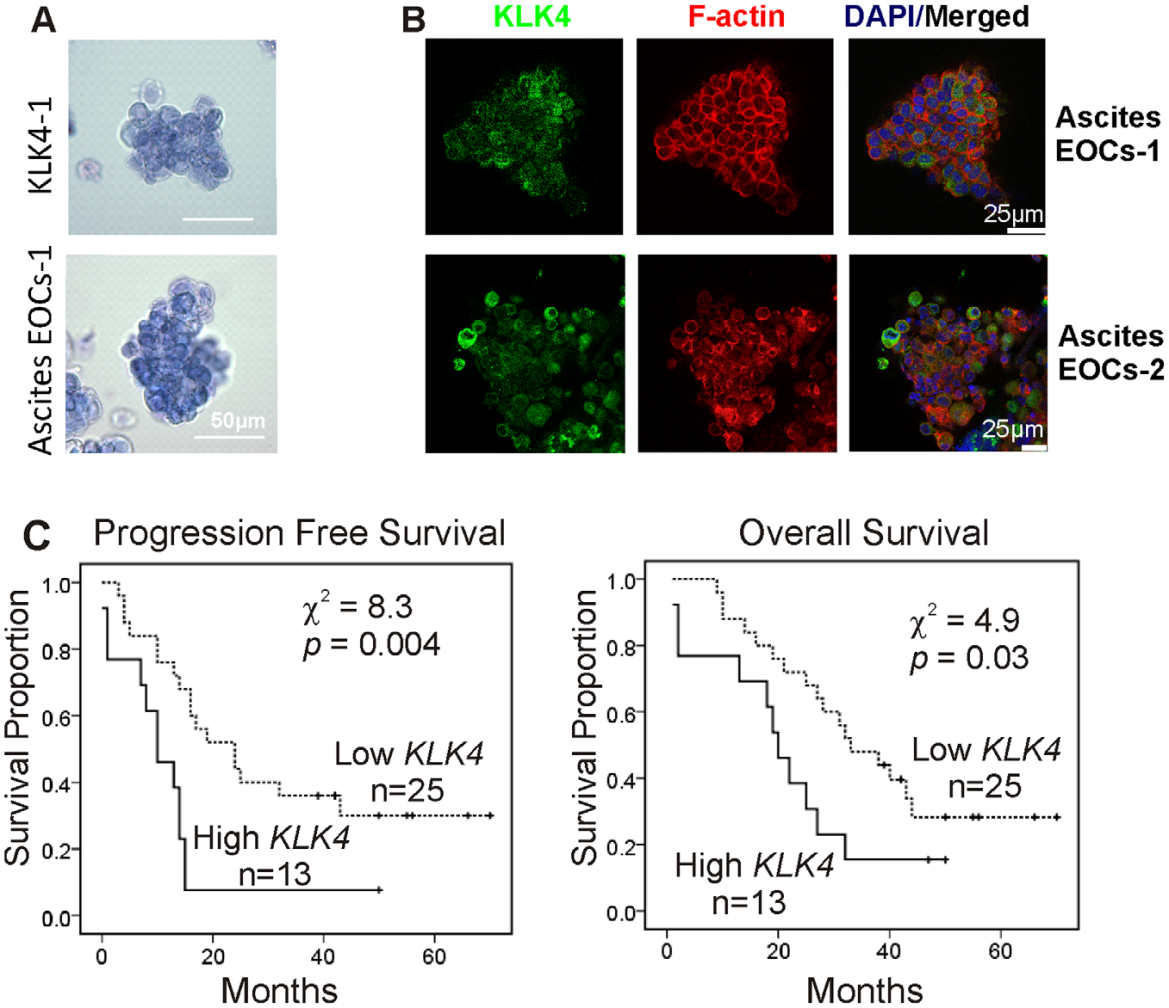
KLK4 is associated with poor outcome of patients. **A.** Phase contrast images show the similar morphology of trypan blue stained KLK4-1 MCAs and ascites-derived MCAs from a serous EOC patient. Scale bar, 50 µm. **B.** IF confocal microscopy with an antibody against KLK4 (green), phalloidin (red) and DAPI (blue) in ascitic serous EOC MCAs from 2 patients; scale bars, 25 µm. **C.** Kaplan-Meier survival analysis shows the relationship between *KLK4* mRNA levels in tumor tissue samples and survival status of a cohort of 38 serous EOC patients. Left panel, progression free survival (PFS) time for patients with low *KLK4* (n = 25) and high *KLK4* (n = 13) levels (χ^2^ = 8.3, *p* = 0.004). Right panel, overall survival time for patients with low *KLK4* (n = 25) and high *KLK4* (n = 13) levels (χ^2^ = 4.9, *p* = 0.03).

To further determine the clinical relevance of KLK4 induced MCAs observed *in vitro*, the morphology of ascitic MCAs from EOC patients was compared with KLK4 transfected cells. Trypan blue stained KLK4-1 MCAs displayed a similar appearance to the EOC spheroids derived from ascites (Fig. 6A). IF microscopy images showed that KLK4 was localized in the cytoplasm of ascites-derived MCAs from 2 patients (Fig. 6B).

To determine the association of high *KLK4* levels with prognosis in EOC specifically, compared to that reported previously for ovarian cancer patients with mixed histotypes [24], [25], we performed RT-qPCR in a cohort of cDNA samples from 38 serous EOC patients. Kaplan-Maier survival analysis showed that the patients with high tumor *KLK4* had significantly shorter progression free (PFS, χ^2^ = 8.3, P = 0.004) and overall survival time (χ^2^ = 4.9, P = 0.03) than those with low *KLK4* (Fig. 6C). Pearson Correlation analysis revealed an association of high *KLK4* levels with late stage (P = 0.017), large residual tumor volume (P = 0.034) and no-response to chemotherapy (P = 0.03) but not the tumor grade (P = 0.95), tumor origin (primary or metastatic sites, P = 0.59) or ascites volume (P = 0.38, Supplementary Table S2).

## Discussion

We report here that KLK4 over-expression promotes MCA/spheroid formation and paclitaxel resistance in a 3D-suspension microenvironment akin to that seen in ascites, and increases spreading of these cells on a mesothelial monolayer, important in EOC invasive behavior. KLK4 inhibition reduced MCA compaction and increased sensitivity to paclitaxel confirming the integral role of KLK4 in these events. Although these data were generated in only two serous ovarian cancer cell lines (SKOV-3 and OVCA432) and may not be representative of all serous EOC, they support our own findings here in a serous EOC cohort, and previous reports in ovarian cancer cohorts of mixed histotypes [24], [25], that women with high *KLK4* mRNA levels in their tumors had shorter progression free and overall survival time. Notably, the latter cohorts consisted of largely serous EOC patients (64/145, ref [24]; 38/46, ref [25]). Overall, these findings indicate that KLK4 not only has potential as a prognostic biomarker, but as a therapeutic target to increase paclitaxel sensitivity for a subgroup of EOC patients with high KLK4 levels.

The host microenvironment reprograms cell phenotypes promoting cancer progression including peritoneal metastasis of EOC [13]. Multicellular aggregation is one such event that aids tumor cells to survive in a 3D-suspension ascites microenvironment *via* reprogramming cellular phenotype and relevant molecules, such as integrins [36], [38]. Our previous study showed that the KLK7 peptidase promotes MCA formation and cell-ECM interaction via α5β1 integrins [23]. However, in contrast to the KLK7-expressing SKOV-3 clones, KLK4-expression in SKOV-3 cells did not increase α5β1 integrin levels compared to the vector/native cells but induced expression of uPA, suggesting different molecular mechanisms are involved. Furthermore, in contrast to the preferable adhesion to fibronectin and vitronectin, but not type I or IV collagen, observed in KLK7-SKOV-3 cells [23] KLK4-SKOV-3 cells did not show increased adhesion to any of these ECM proteins. However, more compact MCAs still formed in the ascites mimicry 3D-suspension microenvironment when KLK4 was over-expressed in, or when active KLK4 was added to, SKOV-3 cells. Like the KLK7 spheroids [23] the KLK4-MCAs were able to invade into mesothelial cell monolayers and produced larger cancer cell foci than those formed by vector and native SKOV-3 cells. Although the less compact Vector-transfected and native SKOV-3 MCAs can also re-grow into mesothelial monolayers, the larger compact KLK4 SKOV-3 MCAs with more cells likely generate more contractile force to drive mesothelial cells apart, mimicking the invasive EOC phenotype as reported previously [31], [39], [40].

Chemoresistance is a major obstacle to conquer in prolonging the survival time of ovarian cancer patients. In addition to the chemoresistance observed with MCAs compared to monolayer cells [41], compact MCAs are less responsive to various treatments including chemotherapies than scattered small ones [42]. The penetrating gradient of cytotoxic drugs has an impact on their uptake by the cells in the centre of MCAs as it does on nutrients and oxygen [42]. That platinum resistance is related to cellular P-glycoprotein level but not the hypoxic status [43] may explain why we saw no difference in cell survival between the compacted KLK4-expressing and scattered control SKOV-3 MCAs on platinum treatment. However, the paclitaxel resistance seen in compact KLK4-MCAs may be associated with hypoxia in the inner cell mass of MCAs [44]. Blocking certain peptidases using a small molecule inhibitor may be a potential adjunct therapeutic strategy as we saw that the addition of a KLK4 inhibitory antibody or selective KLK4 active site inhibitor (SFTI-FCQR) [27] increased paclitaxel sensitivity of MCAs formed by KLK4 expressing cells. Consistent with this observation, adding the general serine protease inhibitor, aprotinin, also showed reduced compaction of KLK4-MCAs and increased response to paclitaxel although to a lesser degree. This suggests a complex interplay of serine proteases such that blocking their activity with aprotinin may in some cases enhance their resistance to paclitaxel. However, our data is consistent with a previous observation in a xenograft mouse model showing that a combined regime of the general Kunitz-type serine protease inhibitor bikunin and paclitaxel reduced intra-peritoneal tumor burden and ascites volume [45]. Interestingly, a previous study showed an association of uPA expression and chemoresistance in ovarian cancer patients [10]. In addition, the clearance of mesothelial monolayers may also reflect a role of uPA secreted by EOC cells in the MCAs or other potential mechanisms yet to be determined. Whether this is a downstream event of KLK4 activation remains to be determined. These data suggest that KLK4 and other KLK serine peptidases, such as KLK7 [23], play a role, at least in part, in MCA related chemoresistance.

KLK4 is synthesized as a pre-pro-enzyme with signal peptide cleavage during secretion; after secretion the pro-region is cleaved to activate KLK4. To date there is no published literature regarding the auto-activation of KLK4 or activation of this enzyme in cancer cells. However, it has been reported that KLK4 can be activated by MMP20 during tooth development in the pig [46], [47] suggesting that MMP20 or an MMP with a similar specificity may activate KLK4 in the ovarian cancer microenvironment. The active KLK4 enzyme can bind to serpins to form a covalent complex, such as α1-antitrypsin and α2-antiplasmin [48]. We have provided evidence that the 88 kDa band observed here may reflect a bound form of KLK4 to a serum binding protein or serpin since the KLK peptidases are known to covalently bind through their active site to serpins [49]. Similarly, our data suggests that the KLK4 secreted by the KLK4-transfected SKOV-3 cells was active since it formed a high molecular weight complex in the presence of serum while the catalytically inactive mutant-KLK4S/A did not form such a complex. Thus, a complex regulatory system, similar to uPA regulation by PAI-1 [50], [51], likely exists for the KLK peptidases in the ovarian cancer ascites microenvironment. Indeed, several KLKs, such as KLK6 and KLK7, have been identified in ascites derived from women with EOC using unbiased proteomic approaches [52], [53]. In addition, KLKs regulate cell signaling pathways *via* extracellular proteolysis [14]. For example, at a biochemical level, active KLK4 cleaves pro-uPA generating active single chain uPA [17] which is induced by ascites in EOC cells [9] and is associated with chemoresistance and poor prognosis in women with this cancer [10], [11]. Furthermore, high levels of KLK5-KLK8 and uPA have been associated with late stage ovarian cancer in patients who have already developed metastasis [54]. Both preclinical *in vitro* and *in vivo* studies revealed that inhibition of tumor associated uPA reduced invasive behaviors of cancer cells [50], but the effects of its inhibitors were minimal in clinical trials [51]. In this study, we showed for the first time that KLK4 induced expression of pro-uPA in SKOV-3 EOC cells providing a possible molecular mechanism by which KLK4 promotes invasive behaviors and chemoresistance via its downstream targets. Although the precise substrate(s) cleaved by KLK4 that are important in the MCA formation and chemoresistance observed here remain to be clarified, pro-uPA is a promising candidate. Our data showing KLK4 expression in ascites-derived EOCs is consistent with a previous clinical observation of high KLK4 levels in EOC samples from patient effusions [55] which further supports our overall findings here, and that of others, that high KLK4 levels are associated with EOC progression and chemoresistance [24], [25]. These findings may also reflect high KLK4 levels in ascites fluid although previous studies showed a low level of KLK4 in ascites or effusions from ovarian cancer patients [56], [57], In the future, it will be important to correlate KLK levels in ascites with that in cells derived from ascites as well as serum to determine the prognostic utility of such information. The concurrent expression of uPA in these cells strongly suggests an association that may involve a hierarchical cascade of serine protease activation. However, the increased blockade of KLK4 action by the selective KLK4 inhibitor, SFTI-FCQR, compared to that seen with aprotinin suggests that other, as yet unidentified, substrates of KLK4 must also be involved.

Our data that adding active rKLK4 significantly increased compaction of MCAs formed by SKOV-3 cells compared to the inactive mutant KLK4S/A and PBS controls support a role for KLK4 enzymatic activity in this process. However, it is notable that when inactive mutant KLK4S/A was added into the SKOV-3 cultures, the MCAs formed were more compact than controls, suggesting that not only the KLK4 enzyme but the inactive KLK4S/A also plays a role in MCA formation. These data indicate that the action of KLK4 in MCA formation involves both proteolytic and nonproteolytic effects. Thus, conceivably the KLK4 functional blocking antibody is also inhibiting non proteolytic KLK4 effects although the data from the use of the KLK4 selective active site SFTI inhibitor clearly indicates that there is a significant proteolytic component to this effect as well. Consistent with this argument, a recent study showed that catalytically inactive KLK10, another member of this gene family, displayed a role as a tumor suppressor on ES2 ovarian cancer cells both *in vitro* and *in vivo* [58]. An earlier study revealed that two prostate specific antigen (PSA, also known as KLK3) blocking antibodies, one inhibiting enzymatic activity and one not affecting catalytic activity, both inhibited the biological function of this kallikrein, suggesting that PSA also has an alternative function via protein-protein interaction [59]. Together, these data may also reflect the evolutionary similarity that these kallikreins share with growth factors like nerve growth factors [60] which may in part explain any spatially induced protein-protein interactions.

In summary, we have provided a mechanism for the functional role of KLK4 in EOC progression by promoting MCA formation and chemoresistance in the 3D-suspension microenvironment seen clinically in ascites. Thus KLK4 inhibition may be a potential enhancer of paclitaxel sensitivity for a subgroup of serous EOC patients with high KLK4 levels.

## Supporting Information

Figure S1 **A.** RT-PCR showing expression of KLK4 (K4Ex1For, 5′-ATGGCCACAGCAGGAAATCCC-3′; K4Ex4Rev, 5′-CACGCACTGCAGCACGGTAG-3′) in EOC cell lines OV90, Caov-3, OVCA420, PEO1, PEO4, PEO14, OVCA432, OAW42 and OVCAR-3, but not mesothelial LP9, normal ovarian epithelial HOSE17.1, EOC SKOV-3, OVMZ-6 or NFF cells with no cDNA as negative control. *β2-microglobulin* (*β2M*) (β2MFor, 5′- TGAATTGCTATGTGTCTGGGT-3′, β2MRev, 5′- CCTCCATGATGCTGCTTACAT-3′) serves as a loading control. **B.** Recombinant active KLK4 bound to unknown serpins/proteins in the FCS containing media. Western blot analysis using anti-V5 primary antibody as described in Materials and Methods showing the 88 kDa protein band formed by recombinant active KLK4 at concentration of 100 nM or 500 nM in the presence of either 1% or 5% FCS containing RPMI-1640 at time points 2 h (Lanes 1–7) and 18 h (Lanes 8–14) respectively. Precision Plus Protein Dual Color Standards #161-0374EDU were from Bio-Rad with molecular weight (kDa) indicated. Lanes 1 and 8, KLK4 500 nM in PBS; 2 and 9, KLK4 100 nM in RPMI-1640; 3 and 10, KLK4 500 nM in RPMI-1640; 4 and 11, KLK4 100 nM in RPMI-1640 with 1% FCS; 5 and 12, KLK4 500 nM in RPMI-1640 with 1% FCS; 6 and 13, KLK4 100 nM in RPMI-1640 with 5% FCS; 7 and 14, KLK4 500 nM in RPMI-1640 with 5% FCS respectively. **C.** Immunofluorescent microscopy confirmed KLK4 protein expression in SKOV-3 cells stably over-expressing KLK4 clones but not in Vector-1, native SKOV-3 control cells or IgG negative control on KLK4-1. KLK4 is in green detected by antibody against V5 or KLK4 and phalloidin staining F-actin in red. Scale bar, 20 µm.
(TIF)

Figure S2 **KLK4 overexpressing SKOV-3 cells are chemoresistant.** Left panel, WST-1 assay shows cell survival after cisplatin treatment in KLK4-SKOV-3 cells compared to vector control cells in 2D-monolayer conditions (72 h treatment), *P<0.05. Right panel, KLK4-SKOV-3 cells appear resistant to paclitaxel in 2D-monolayer culture but this does not reach significance. Mean ± SEM of 3 KLK4/vector-SKOV-3 clones from 3 separate experiments in triplicates.
(TIF)

Table S1 **Clinical, pathological characteristics and** ***KLK4*** **levels of patients.** ^1^Patients were classified into 3 groups based on chemotherapy response and clinical observations. ^2^WD, MD, PD = well, moderately or poorly differentiated, U = unknown. ^3^G = gross amount of tissue remaining. ^4^1° = cancerous ovarian tissue, 2° = diseased adjacent metastatic tissue. Ascites: L = Large, >5,000 ml; M = med, 1,000–5,000 ml; S = small, <1,000 ml; or 0 = nil; U = unknown. ^5^Chemo-treatment: C = carboplatin, T = Taxol, CT = carboplatin and taxol, CTG = carboplatin, taxol and gemcitabine, clinical trial, U = unknown; Chemotherapy notes: ^6^patient had 3 cycles of chemotherapy pre-surgery to reduce tumor load, then had normal chemotherapy after surgery. ^7^Patient had 2 cycles and died. ^8^N = no chemotherapy, patient died with brain metastases. ^9^Patient had previous surgery, no previous chemotherapy. ^10^Recurerent cancer, original chemotherapy was CT. ^11^CT for 1 cycle then carboplatin only for 5 cycles. ^12^Progression free survival. ^13^ *KLK4* levels in tumor samples, H = high, L = low. ^14^Y = deceased, N = alive.
(DOC)

Table S2 **Relationship between** ***KLK4*** **levels and clinical parameters in 38 serous EOC patients.** *One way Pearson analysis was performed for association of KLK4 levels and stage, grade, residual tumor size, origin, ascites volume and chemo-response respectively. *p*<0.05 is statistically significant.
(DOC)
