# Supplementary figures and images for: Paclitaxel Resistance and Multicellular Spheroid Formation Are Induced by Kallikrein-Related Peptidase 4 in Serous Ovarian Cancer Cells in an Ascites Mimicking Microenvironment
Source: PLoS One. 2013 Feb 25;8(2):e57056. doi: 10.1371/journal.pone.0057056 (PMC3581584; doi:10.1371/journal.pone.0057056)

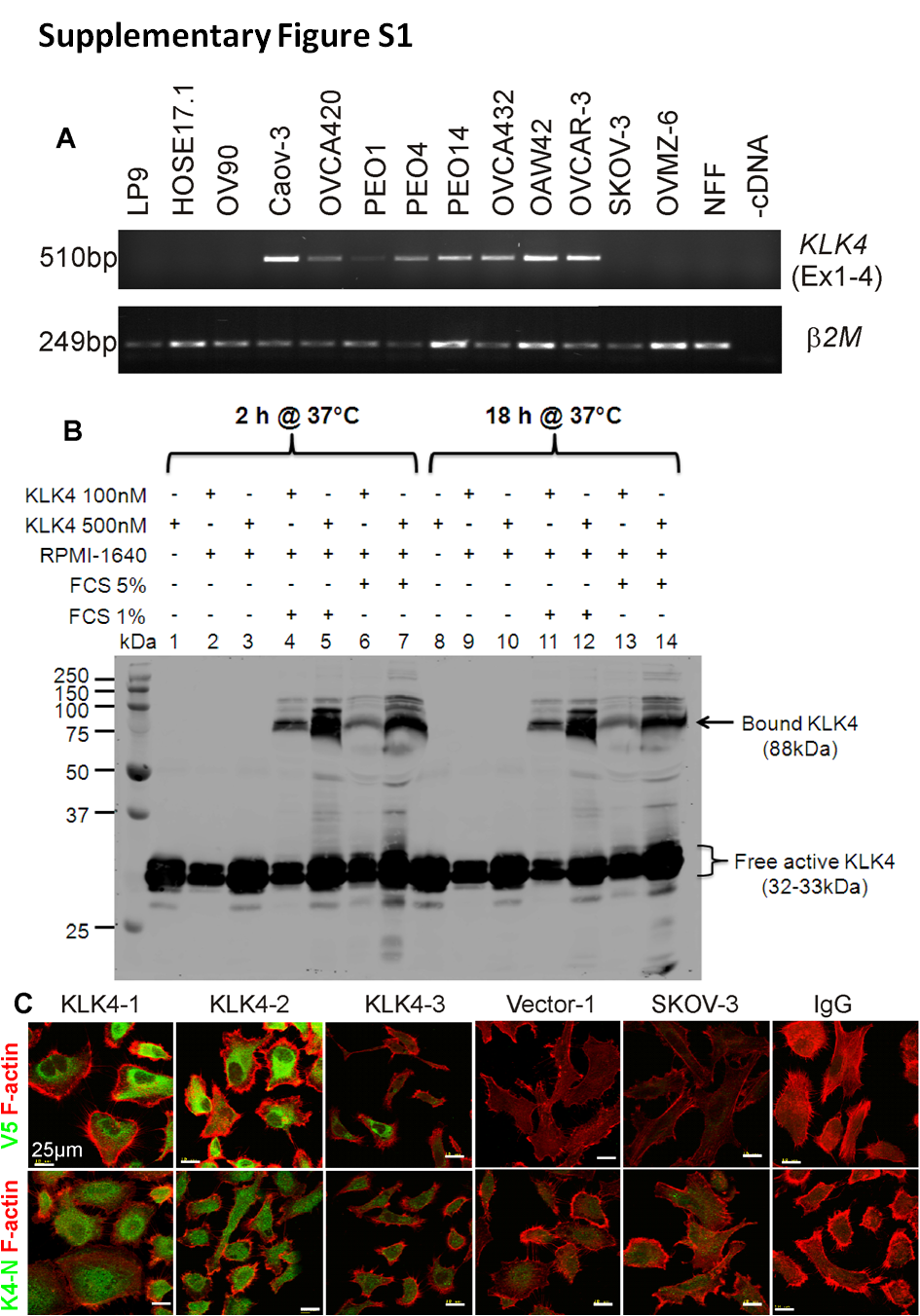

Supplement: Figure S1 — A. RT-PCR showing expression of KLK4 (K4Ex1For, 5′-ATGGCCACAGCAGGAAATCCC-3′; K4Ex4Rev, 5′-CACGCACTGCAGCACGGTAG-3′) in EOC cell lines OV90, Caov-3, OVCA420, PEO1, PEO4, PEO14, OVCA432, OAW42 and OVCAR-3, but not mesothelial LP9, normal ovarian epithelial HOSE17.1, EOC SKOV-3, OVMZ-6 or NFF cells with no cDNA as negative control. β2-microglobulin (β2M) (β2MFor, 5′- TGAATTGCTATGTGTCTGGGT-3′, β2MRev, 5′- CCTCCATGATGCTGCTTACAT-3′) serves as a loading control. B. Recombinant active KLK4 bound to unknown serpins/proteins in the FCS containing media. Western blot analysis using anti-V5 primary antibody as described in Materials and Methods showing the 88 kDa protein band formed by recombinant active KLK4 at concentration of 100 nM or 500 nM in the presence of either 1% or 5% FCS containing RPMI-1640 at time points 2 h (Lanes 1–7) and 18 h (Lanes 8–14) respectively. Precision Plus Protein Dual Color Standards #161-0374EDU were from Bio-Rad with molecular weight (kDa) indicated. Lanes 1 and 8, KLK4 500 nM in PBS; 2 and 9, KLK4 100 nM in RPMI-1640; 3 and 10, KLK4 500 nM in RPMI-1640; 4 and 11, KLK4 100 nM in RPMI-1640 with 1% FCS; 5 and 12, KLK4 500 nM in RPMI-1640 with 1% FCS; 6 and 13, KLK4 100 nM in RPMI-1640 with 5% FCS; 7 and 14, KLK4 500 nM in RPMI-1640 with 5% FCS respectively. C. Immunofluorescent microscopy confirmed KLK4 protein expression in SKOV-3 cells stably over-expressing KLK4 clones but not in Vector-1, native SKOV-3 control cells or IgG negative control on KLK4-1. KLK4 is in green detected by antibody against V5 or KLK4 and phalloidin staining F-actin in red. Scale bar, 20 µm. (TIF) [file pone.0057056.s001.tif]
