# Supplementary material for: Paclitaxel Resistance and Multicellular Spheroid Formation Are Induced by Kallikrein-Related Peptidase 4 in Serous Ovarian Cancer Cells in an Ascites Mimicking Microenvironment
Source: PLoS One. 2013 Feb 25;8(2):e57056. doi: 10.1371/journal.pone.0057056 (PMC3581584; doi:10.1371/journal.pone.0057056)
Supplement: Table S1 — Clinical, pathological characteristics and KLK4 levels of patients. 1Patients were classified into 3 groups based on chemotherapy response and clinical observations. 2WD, MD, PD = well, moderately or poorly differentiated, U = unknown. 3G = gross amount of tissue remaining. 41° = cancerous ovarian tissue, 2° = diseased adjacent metastatic tissue. Ascites: L = Large, >5,000 ml; M = med, 1,000–5,000 ml; S = small, <1,000 ml; or 0 = nil; U = unknown. 5Chemo-treatment: C = carboplatin, T = Taxol, CT = carboplatin and taxol, CTG = carboplatin, taxol and gemcitabine, clinical trial, U = unknown; Chemotherapy notes: 6patient had 3 cycles of chemotherapy pre-surgery to reduce tumor load, then had normal chemotherapy after surgery. 7Patient had 2 cycles and died. 8N = no chemotherapy, patient died with brain metastases. 9Patient had previous surgery, no previous chemotherapy. 10Recurerent cancer, original chemotherapy was CT. 11CT for 1 cycle then carboplatin only for 5 cycles. 12Progression free survival. 13 KLK4 levels in tumor samples, H = high, L = low. 14Y = deceased, N = alive. (DOC) [file pone.0057056.s003.doc]

**Supplementary Table S1. Clinical, pathological characteristics and *KLK4* levels of patients.**

| Chemotherapy  Response^1^ | Patient  ID | FIGO stage | Grade^2^ | Residual (cm)^3^ | Specimen Origin^4^ | Ascites (ml) | Chemo  Tmt^5^ | PFS^12^  Months | Survival  Months | KLK4^13^ | Deceased^14^ |
| --- | --- | --- | --- | --- | --- | --- | --- | --- | --- | --- | --- |
| Non-Responder | 1 | IIC | PD | <1 | 1º | S | C | 1 | 2 | H | Y |
|  | 2 | IIIC | PD | G^3^ | 2º | 0 | CT^6^ | 5 | 14 | L | Y |
|  | 3 | IIIC | MD | <1 | 1º | L | U^7^ | 0 | 2 | H | Y |
|  | 4 | IIIC | PD | <1 | 1º | M | CT | 2 | 9 | H | Y |
|  | 5 | IIIC | U | G | 2º | S | CT | 3 | 13 | H | Y |
|  | 6 | IIIC | PD | 1-2 | 1º | S | CT | 3 | 10 | L | Y |
|  | 7 | IIIC | PD | 1-2 | 2º | L | CT | 14 | 16 | L | Y |
|  | 8 | IIIC | PD | G | 2º | M | C | 5 | 19 | H | Y |
|  | 9 | IIIC | PD | G | 2º | L | CT | 5 | 19 | H | Y |
|  | 10 | IV | PD | 0 | 1º | 0 | N^8^ | 0 | 1 | H | Y |
|  | 11 | IV | PD | <1 | 2º | M | CT | 3 | 10 | L | Y |
|  |  |  |  |  |  |  |  |  |  |  |  |
| Responder, | 12 | IIIC | MD | <1 | 2º | L | CT | 10 | 32 | H | Y |
| Relapse less | 13 | IIIC | MD | G | 2º | S | CT | 15 | 18 | H | Y |
| than 12 months | 14 | IIIC | MD | <1 | 2º | S | CT | 14 | 27 | L | Y |
|  | 15 | IIIC | PD | 1-2 | 2º | S | CT | 10 | 33 | L | Y |
|  | 16 | IIIC | PD | <1 | 2º | M | CT^9^ | 16 | 28 | L | Y |
|  | 17 | IIIC | PD | G | 2º | U | CT | U | >47 | H | N |
|  | 18 | IIIC | PD | G | 2º | L | CT | 13 | 31 | L | Y |
|  | 19 | IIIC | PD | G | 1º | 0 | CT^10^ | 24 | 25 | H | Y |
|  | 20 | IV | PD | G | 1º | L | CT | 88 | 22 | H | Y |
|  |  |  |  |  |  |  |  |  |  |  |  |
| Responder | 21 | IIB | MD | 0 | 1º | 0 | CT | 13 | 22 | L | Y |
| No Relapse | 22 | IIIB | PD | <1 | 1º | 0 | CT | 24 | 38 | L | Y |
| At 12 months | 23 | IIIB | WD | 1-2 | 1º | 0 | CT | U | >162 | L | Y |
|  | 24 | IIIC | PD | 1-2 | 2º | L | CT | U | >56 | L | N |
|  | 25 | IIIC | PD | <1 | 2º | S | CT | U | >55 | L | N |
|  | 26 | IIIC | MD | 1-2 | 1º | S | CT | 17 | 44 | L | Y |
|  | 27 | IIIC | PD | <1 | 2º | S | CT^11^& C | 32 | 40 | L | Y |
|  | 28 | IIIC | PD | 1-2 | 1º | M | CT | U | >50 | H | N |
|  | 29 | IIIC | U | U | 1º | U | CT | U | >50 | L | N |
|  | 30 | IIIC | PD | G | 2º | 0 | CT | U | >43 | L | N |
|  | 31 | IIIC | PD | G | 2º | M | CT | 25 | 27 | L | Y |
|  | 32 | IIIC | PD | <1 | 2º | 0 | CT | U | >42 | L | N |
|  | 33 | IIIC | PD | 1-2 | 2º | 0 | CT | U | >42 | L | N |
|  | 34 | IIIC | PD | 0 | 1º | 0 | T | 16 | 32 | L | Y |
|  | 35 | IIIC | PD | 0 | 2º | 0 | CTG | U | >39 | L | N |
|  | 36 | IIIC | PD | G | 1º | M | CT | 14 | 25 | L | Y |
|  | 37 | IIIC | WD | 1-2 | 1º | S | CT | U | >66 | L | Y |
|  | 38 | IIIC | PD | G | 1º | M | CC | 19 | 22 | L | Y |

^1^Patients were classified into 3 groups based on chemotherapy response and clinical observations. ^2^WD, MD, PD = well, moderately or poorly differentiated, U = unknown. ^3^G = gross amount of tissue remaining. ^4^1º = cancerous ovarian tissue, 2º = diseased adjacent metastatic tissue. Ascites: L = Large, >5,000 ml; M = med, 1,000-5,000 ml; S = small, <1,000 ml; or 0 = nil; U = unknown. ^5^Chemo-treatment: C = carboplatin, T = Taxol, CT = carboplatin and taxol, CTG = carboplatin, taxol and gemcitabine, clinical trial, U = unknown; Chemotherapy notes: ^6^patient had 3 cycles of chemotherapy pre-surgery to reduce tumor load, then had normal chemotherapy after surgery. ^7^Patient had 2 cycles and died. ^8^N = no chemotherapy, patient died with brain metastases. ^9^Patient had previous surgery, no previous chemotherapy. ^10^Recurerent cancer, original chemotherapy was CT. ^11^CT for 1 cycle then carboplatin only for 5 cycles. ^12^Progression free survival. ^13^*KLK4* levels in tumor samples, H = high, L = low. ^14^Y = deceased, N = alive.

**Supplementary Table S2. Relationship between *KLK4* levels and clinical parameters in 38 serous EOC patients.**

| Variables | Patients | No. of patients | | *p* value |
| --- | --- | --- | --- | --- |
|  |  | Low *KLK4* | High *KLK4* |  |
| Stage | 38 | 25 | 13 | 0.017* |
| II | 2 | 1 | 1 |  |
| III | 33 | 23 | 10 |  |
| IV | 3 | 1 | 2 |  |
|  |  |  |  |  |
| Grade | 38 | 25 | 13 | 0.95 |
| Well | 2 | 2 | 0 |  |
| Mod | 6 | 3 | 3 |  |
| Poor | 28 | 19 | 9 |  |
| Unknown | 2 | 1 | 1 |  |
|  |  |  |  |  |
| Residual tumor (cm) | 38 | 25 | 13 | 0.034* |
| 0 | 4 | 4 | 0 |  |
| ≤ 1 | 11 | 8 | 3 |  |
| 1-2 | 9 | 5 | 4 |  |
| Gross | 13 | 7 | 6 |  |
| Unknown | 1 | 1 |  |  |
|  |  |  |  |  |
| Tumour origin | 38 | 25 | 13 | 0.59 |
| Primary | 17 | 14 | 3 |  |
| Metastasis | 21 | 11 | 10 |  |
|  |  |  |  |  |
| Ascites | 38 | 25 | 13 | 0.38 |
| Nil | 11 | 9 | 2 |  |
| Small | 10 | 7 | 3 |  |
| Med | 8 | 5 | 3 |  |
| Large | 7 | 3 | 4 |  |
| Unknown | 1 |  | 1 |  |
|  |  |  |  |  |
| Chemotherapy response | 38 | 25 | 13 | 0.03* |
| Non-Response | 11 | 4 | 7 |  |
| Responder, Relapse < 12mth | 9 | 4 | 5 |  |
| Responder, Relapse ≥12mth | 18 | 17 | 1 |  |

*One way Pearson analysis was performed for association of KLK4 levels and stage, grade, residual tumor size, origin, ascites volume and chemo-response respectively. *p* < 0.05 is statistically significant.
