# Supplementary material for: Paclitaxel Resistance and Multicellular Spheroid Formation Are Induced by Kallikrein-Related Peptidase 4 in Serous Ovarian Cancer Cells in an Ascites Mimicking Microenvironment
Source: PLoS One. 2013 Feb 25;8(2):e57056. doi: 10.1371/journal.pone.0057056 (PMC3581584; doi:10.1371/journal.pone.0057056)
Supplement: Table S2 — Relationship between KLK4 levels and clinical parameters in 38 serous EOC patients. *One way Pearson analysis was performed for association of KLK4 levels and stage, grade, residual tumor size, origin, ascites volume and chemo-response respectively. p<0.05 is statistically significant. (DOC) [file pone.0057056.s004.doc]

**Supplementary Table S2. Relationship between *KLK4* levels and clinical parameters in 38 serous EOC patients.**

| Variables | Patients | No. of patients | | *p* value |
| --- | --- | --- | --- | --- |
|  |  | Low *KLK4* | High *KLK4* |  |
| Stage | 38 | 25 | 13 | 0.017* |
| II | 2 | 1 | 1 |  |
| III | 33 | 23 | 10 |  |
| IV | 3 | 1 | 2 |  |
|  |  |  |  |  |
| Grade | 38 | 25 | 13 | 0.95 |
| Well | 2 | 2 | 0 |  |
| Mod | 6 | 3 | 3 |  |
| Poor | 28 | 19 | 9 |  |
| Unknown | 2 | 1 | 1 |  |
|  |  |  |  |  |
| Residual tumor (cm) | 38 | 25 | 13 | 0.034* |
| 0 | 4 | 4 | 0 |  |
| ≤ 1 | 11 | 8 | 3 |  |
| 1-2 | 9 | 5 | 4 |  |
| Gross | 13 | 7 | 6 |  |
| Unknown | 1 | 1 |  |  |
|  |  |  |  |  |
| Tumour origin | 38 | 25 | 13 | 0.59 |
| Primary | 17 | 14 | 3 |  |
| Metastasis | 21 | 11 | 10 |  |
|  |  |  |  |  |
| Ascites | 38 | 25 | 13 | 0.38 |
| Nil | 11 | 9 | 2 |  |
| Small | 10 | 7 | 3 |  |
| Med | 8 | 5 | 3 |  |
| Large | 7 | 3 | 4 |  |
| Unknown | 1 |  | 1 |  |
|  |  |  |  |  |
| Chemotherapy response | 38 | 25 | 13 | 0.03* |
| Non-Response | 11 | 4 | 7 |  |
| Responder, Relapse < 12mth | 9 | 4 | 5 |  |
| Responder, Relapse ≥12mth | 18 | 17 | 1 |  |

*One way Pearson analysis was performed for association of KLK4 levels and stage, grade, residual tumor size, origin, ascites volume and chemo-response respectively. *p* < 0.05 is statistically significant.
